# Novel Antibacterial Agents 2022

**DOI:** 10.3390/ph17030370

**Published:** 2024-03-13

**Authors:** Fiorella Meneghetti, Daniela Barlocco

**Affiliations:** Department of Pharmaceutical Sciences, University of Milan, Via L. Mangiagalli 25, 20133 Milan, Italy; daniela.barlocco@unimi.it

This Special Issue contains 16 original articles, 3 reviews, and 1 communication. The authors of these papers focused on the topic of multidrug-resistant (MDR) bacteria and addressed several targets either by natural or synthetic compounds as well as novel techniques.

In particular, in the field of natural compounds, Abdulrahman S. Bazaid et al. identified, by GC-MS analysis, 2,4-dihydroxy-2,5-dimethyl-3(2*H*)-furan-3-one and 1-methylcyclopropanemethanol as the major phytoconstituents of Sumra honey [1] and suggested that this source be used as a promising potential therapeutic tool against infections caused by MDR bacteria and fungi [2]. In addition, they proposed Sumra honey as a good candidate to inhibit bacterial cellular communication in strains of *P. aeruginosa* and *C. violaceum* [1].

Zenon Węglarz et al. studied the chemical profile of the species cultivated in the temperate climate of Central Europe of Helichrysum italicum (Roth) G. Don., one of the most important medicinal plants originating from the Mediterranean region of Europe [3]. Both herbs and inflorescences were analyzed. Neryl acetate, accompanied by α-pinene in the herb (10.42%), and nerol in inflorescences (15.73%), were the most important components. When tested for their antioxidant properties, both methanolic extract and essential oil obtained from the herb indicated a higher potential than those originating from the inflorescences. By contrast, the antimicrobial activity of the essential oil from inflorescences was higher than that of the herb essential oil. Gram-positive bacteria were more sensitive to both essential oils in comparison with Gram-negative bacteria [4].

The antibacterial activity of a <10 kDa peptide-rich extract obtained through the autolysis of yeast biomass under mild thermal treatment with self-proteolysis by endogenous peptidases was reported [5]. Maria Fernanda da Silva Santos et al., by in silico analysis using four independent algorithms, identified fifty-eight AMP candidate sequences which possibly contribute to the bacterial inactivation; then, they recommended *S. cerevisiae*-biomass peptides as promising adjuvants to treat infectious diseases that are poorly sensitive to conventional antibiotics, as previously proposed [6].

Tafenoquine and a derivative of chondrofoline [7,8] were tested for their antileishmanial activity against *L. tropica* (HTD7) by Sayyed Ibrahim Shah et al. In vitro tests (intra-THP-1 amastigote activity) showed that 10-hydroxy chondrofoline was more potent than tafenoquine (LD50 43.80 µM and 53.57 µM, respectively, after 48 h of incubation).

Varsha Srivastava et al. evaluated the antimycobacterial activity of the extracts of *S. xanthocarpum* Schrad. & Wendl., authenticated according to the Ayurvedic Pharmacopoeia of India, against *M. avium* subspecies *paratuberculosis* (MAP) infection [9]. The best inhibition was shown by the hydro-alcoholic extract.

Tuberculosis was the topic of two other articles, which addressed different mycobacterial enzymes.

The enoyl-acyl carrier protein reductase InhA of *M. tuberculosis* is a recognized druggable target [10]. Fawzia Faleh Albelwi et al., based on data from the literature, synthesized a series of 1,2,3-triazole linked to different acetamide groups as inhibitors of InhA. In vitro tests proved that the compounds were able to completely inhibit InhA at a concentration of 10 µM, being better inhibitors than Rifampicin.

Matteo Mori et al., based on their previous results, tested a series of analogues of the 5-(3-cyanophenyl)furan-2-carboxylic acid, the most potent salicylate synthase MbtI inhibitor identified to date [11,12]. Structure–activity relationships evidenced the importance of the side chain linked to the phenyl moiety to improve the in vitro antimycobacterial activity. They also set up a fluorescence-based screening test, using MPI-2 murine cells, which potentially accelerates the identification of new anti-TB drugs.

In recent years, the repurposing of well-known drugs has been proposed as a promising and less-demanding approach in antibiotic research.

On these premises, Tsung-Ying Yang et al. published two papers, investigating the immunomodulator ammonium trichloro(dioxoethylene-O,O′)tellurate (AS101) [13] as an antimicrobial agent.

In the first article, the compound was evaluated against carbapenem-resistant *A. baumannii* (CRAB) [14]. Values of MIC below 50% cytotoxicity were recorded (0.5 to 32 µg/mL and ~150 µg/mL, respectively). The compound displayed better effects than colistin in the carbapenemase-producing *A. baumannii* mouse sepsis model. The accumulation of ROS and disruption of the cell membrane were indicated as antibacterial mechanisms.

Positive results for AS101 were also reported in the second paper, when the compound was tested against colistin- and carbapenem-resistant *K. pneumoniae* (CRKP).

The hybridization of known scaffolds has also provided good results in the identification of new potential drugs [15]. This approach was followed by Riham M. Bokhtia, who synthesized 31 linezolid conjugates and tested them against different strains of bacteria. The most promising agent showed MIC 4.5 µM against *S. aureus* and 2.25 µM against *B. subtilis*. Based on their results, the group developed a robust QSAR (R2 = 0.926, 0.935; R2cvOO = 0.898, 0.915; R2cvMO = 0.903, 0.916 for the *S. aureus* and *B. subtilis* models, respectively) and 3D pharmacophore models.

Several novel synthetic moieties were reported by different groups.

Anthi Petrou et al., following a previous approach, tested seventeen (*Z*)-methyl 3-(4-oxo-2-thioxothiazolidin-5-ylidene)methyl)-1*H*-indole-2-carboxylate derivatives against eight Gram-positive and Gram-negative bacteria [16]. Their activity was found to be higher than that of ampicillin and streptomycin by 10–50-fold. They also displayed significant antifungal activity, with *T. viride* being the most sensitive, while *A. fumigatus* was the most resistant one.

Kevin Schindler et al. presented the results of the computationally evaluated binding affinity of a series of rhenium di- and tricarbonyl diamine complexes against the published structurally characterized membrane-bound *S. aureus* proteins [17]. Two possible major targets were proposed, namely lipoteichoic acids flippase (LtaA) and lipoprotein signal peptidase II (LspA).

Beatriz Suay-García et al. selected two quinolones by applying to a library of 1000 quinolones [18,19] their prediction model of activity against *E. coli*, and they tested them for their antibacterial properties together with a series of zwitterionic quinolonates by a microdilution method. The two quinolones showed the best broad-spectrum activity, though all the compounds were provided with antibacterial properties.

Bacteria resistance was also the topic of three different reviews.

Luigi Principe et al. devoted their attention to the combination of β-lactam/β-lactamase inhibitors (Bls/BLIs) and considered the following associations of drugs: aztreonam/avibactam, cefepime/enmetazobactam, cefepime/taniborbactam, cefepime/zidebactam, cefiderocol, ceftaroline/avibactam, ceftolozane/tazobactam, ceftazidime/avibactam, imipenem/relebactam, meropenem/ nacubactam, and meropenem/vaborbactam [20].

Anika Rütten et al. reported on recent data in the literature concerning bioactivity-based screening methods, focusing on the most relevant assays for the identification of antibiotic activity [21], and mechanisms of action investigations. Successful examples were also reported.

Sekar Madhu et al. focused on recent developments in electrochemical sensing techniques used to assess latent antibiotic resistances of pathogenic bacteria. They highlighted the prevalence of biorecognition probes and tailor-made nanomaterials in electrochemical antibiotic susceptibility testing (AST) [22].

Nanomaterials were also investigated by two other groups.

Maider Ugalde-Arbizu et al. synthesized hybrid nanosystems based on mesoporous silica nanoparticles (MSNs) [23] functionalized with a nicotinic ligand and silver chloride nanoparticles, both phenytoin sodium (Ph)-loaded and unloaded. Their antibacterial activity was evaluated against three different strains of *P. aeruginosa*. The Ph-loaded materials promoted a quasi-complete inhibition of bacterial growth.

Noura Hazime et al. synthesized, and tested against *E. coli*, 184 novel formulations, based on colistin loaded on alginate nanoparticles (Alg Nps) [24], either in the absence or presence of small molecules such as components of essential oils, polyamines, and lactic acid. The formulations, whose safety towards eukaryotic HT-29 cells was established in vitro, are thought to permeabilize the bacterial membrane and cause the leakage of intracellular proteins [25].

Frida Svanberg Frisinger et al. investigated the prototype drug (MAC13243), which interferes with the Gram-negative outer membrane protein LolA on the fecal microbiota. The compound exhibited the concentration-dependent killing of coliforms in two fecal suspensions of healthy donors after 8 h, thus assuring a low risk of inducing dysbiosis.

Finally, a communication by Marius Seethaler et al. proposed a simple one-pot synthesis of fluorinated benzothiophene–indole hybrids as a promising strategy for the search of novel antimicrobial agents [26]. Compounds were evaluated against various *S. aureus* and MRSA strains. Bacterial pyruvate kinase was found to be their molecular target.
